# Supplementary material for: A nonsense mutation of bone morphogenetic protein-15 (BMP15) causes both infertility and increased litter size in pigs
Source: BMC Genomics. 2021 Jan 7;22:38. doi: 10.1186/s12864-020-07343-x (PMC7792226; doi:10.1186/s12864-020-07343-x)
Supplement: Supplementary file 3 — Additional file 3. [file 12864_2020_7343_MOESM3_ESM.pdf]

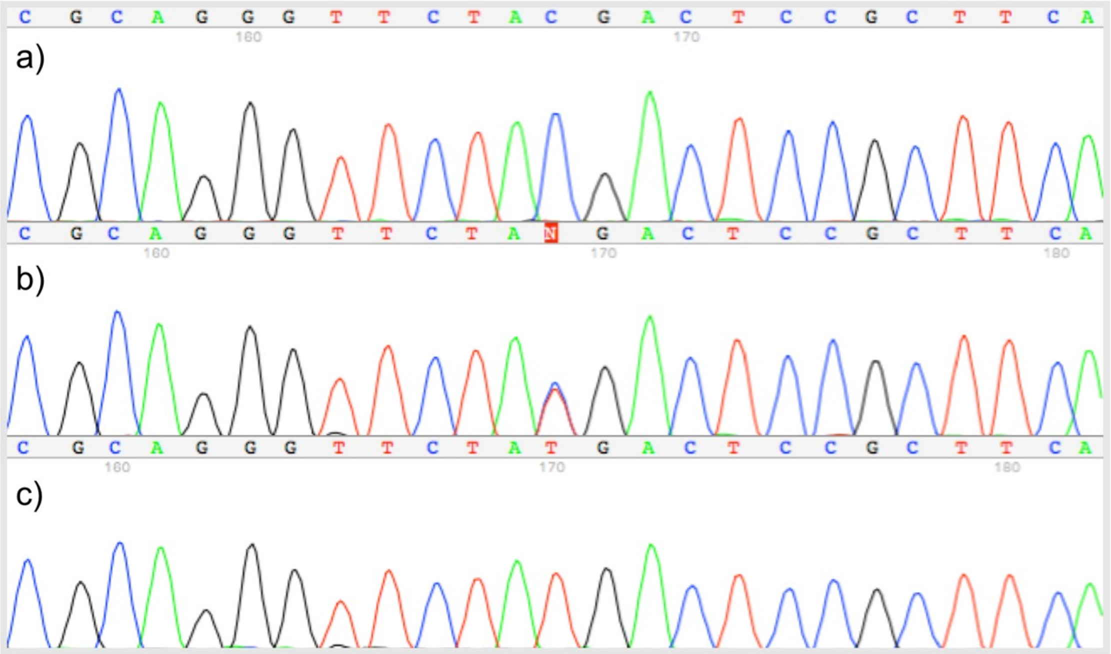

Chromatogram from Sanger-sequencing. a) homozygous for the wild type allele (C/C); b) heterozygous (C/T); c) homozygous for the mutant allele (T/T)
